# Supplementary material for: Are carbon emissions trading and green financial instruments synergistic? -Comprehensive quantitative research based on content analysis
Source: PLoS One. 2024 Mar 7;19(3):e0298601. doi: 10.1371/journal.pone.0298601 (PMC10919601; doi:10.1371/journal.pone.0298601)
Supplement: S1 Data — (ZIP) [file pone.0298601.s001.zip › Data/地级市Z_F工作报告.zip!/┴╔─■/┼╠╜⌡╩╨/└┤╫╘╣1⁄2╓┌║┼í╛┬φ┐╦╩2╛▌═°í┐_2013.docx]

各位代表：

现在，我代表市人民政府，向大会报告工作，请予审议，并请市政协委员和其他列席同志提出意见。

一、2012年工作回顾

刚刚过去的一年，在中共盘锦市委的正确领导下，全市人民团结奋斗，开拓创新，真抓实干，完成了市六届人大三次会议确定的各项任务，书写了新的篇章！

（一）综合实力跃上新台阶

——地区生产总值完成1300亿元，增长12%。

——固定资产投资1000亿元，增长30%。

——公共财政预算收入140.2亿元，增长24.9%。

——社会消费品零售总额256亿元，增长17%。

——外贸出口6.7亿美元，增长24.5%。

——城市居民人均可支配收入27900元，增长15%。

——农民人均纯收入13400元，增长17.5%。

这里，我还要特别报告的是，在国际经济持续低迷、国内经济增长放缓、区域竞争日益激烈的形势下，全市实际利用外资仍达到16.1亿美元，总量全省第三；引进内资400亿元，增速全省第一。

实践证明，任何困难都挡不住盘锦人民前进的步伐，一个崭新的滨海新盘锦正在阔步前进！

（二）项目建设取得新成效

1.一批大项目纷纷落户。新引进亿元以上项目135个，其中10亿元以上项目44个。主要有：

——总投资60亿元的浙江新和成蛋氨酸；

——总投资6亿美元的台湾联成苯酐增塑剂；

——总投资13亿元的航天晨光化工装备；

——总投资10亿元的海德鑫醋酸仲丁酯；

——总投资100亿元的武汉华氏城市综合体；

——总投资6亿美元的北京泛华城市综合体；

——总投资50亿元的新湖中宝国际商贸城；

——总投资50亿元的香港翔球商业综合体；

——总投资50亿元的广东富盈温泉城；

——总投资12亿元的辽宁田园现代农业产业园。

2.一批大项目相继开工。新开工亿元以上项目160个，在建项目290个，其中10亿元以上项目79个。主要有：

——总投资160亿元的长春石化基地；

——总投资32亿元的胜友顺丁橡胶；

——总投资2.5亿美元的戴纳索丁苯橡胶；

——总投资100亿元的华发新城；

——总投资50亿元的苏宁广场；

——总投资48亿元的东方银座中心城；

——总投资23亿元的天沐温泉城；

——总投资20亿元的红星国际广场；

——总投资20亿元的蓝碧源五金机电汽配城；

——总投资12亿元的德胜丰润农产品加工。

3.一批大项目陆续竣工。建成亿元以上项目85个，其中10亿元以上项目12个。主要有：

——和运新材料6万吨丁基橡胶；

——辽河石化60万吨连续重整；

——北燃100万吨灵活多效催化裂化；

——金田8.4万吨双向拉伸聚丙烯薄膜；

——台湾见龙16万吨发泡聚苯乙烯；

——徐工建筑升降机生产基地；

——22万平方米的鹏欣水游城；

——18万平方米的大商商业综合体；

——12万平方米的中国食品城。

这些大项目，是盘锦走向未来、加快发展的重要支撑！

（三）三次产业开创新局面

1.工业经济在应对挑战中加快发展。“工业年”活动成效显著，工业增加值增速全省第一，石化及精细化工产业集群成为我省第一个千亿级产业集群。辽河油田、 北方华锦、辽河石化等大企业对经济增长作出重大贡献。辽东湾石化及精细化工产业园、石油天然气装备制造基地成为国家级新型工业化产业示范基地。辽河重工 CP-300钻井平台交付使用，高新技术产品增加值增长30%。民营经济增加值增长25.9%。

2.服务业发展步伐加快。“金廊银带”跻身辽宁旗舰型服务业集聚区行列，兴隆台中央商务区建设全面推进。旅游业快速发展，湿地观光、温泉度假等特色旅游持 续火爆，旅游总收入增长30%。会展经济发展势头强劲，成功举办中国石油与海洋工程装备博览会、优质稻米交易会、湿地旅游周等大型展会活动。举办5次大型 房交会，成交量2.3万套。商品房销售额增长15.9%。物流、金融、文化等产业不断壮大。

3.农业现代化进程加快。农业机械化水平显著提高，粮食产量达到115.6万吨，再创历史新高。河蟹产量达到6.1万吨，居全国地级市之首。肉蛋奶产量达 到52万吨。隆重举行大米、河蟹展销活动，盘锦大米、河蟹在国内外进一步叫响了品牌。一批国家级农事企业落户盘锦，市级以上龙头企业达到117家。土地流 转面积达到37.5万亩。高升经济区成为国家级现代农业综合示范区。

（四）辽东湾新区建设取得新进展

盘锦港30万吨原油码头及25万吨航道工程启动实施，5个5万吨级泊位投入使用，建成疏港铁路，港口吞吐量达到2000万吨。长春石化、联成化学、宝来石 化、和运橡胶、忠旺铝材、益海嘉里物流等项目加快建设。道路、管网、绿化、填海造地等基础设施工程快速推进，体育中心、学校、医院等重点工程全面进入收尾 阶段。大连理工大学正式落户。辽东湾新区已经成为国家级开发区，成为海峡两岸经贸合作示范区。

（五）城乡一体化发展迈出新步伐

——高标准编制城市规划，明确了城市功能定位、空间布局、一体化发展、生态建设等一系列重大问题，“5城7镇”的大城市框架全面拉开，盘锦走上了建设现代化生态城市的新征程。

——实施城镇大改造、大建设，全市商业、旅游、温泉、城市综合体等大项目开工面积达到1419万平方米，一批标志性建筑拔地而起，城市面貌变得更加美丽！

——加大基础设施建设力度，中华路全线贯通，沈盘铁路、滨海公路、辽东湾互通立交、盘营客专、阜盘高速、兴辽路等交通工程加快推进，华润城市热电开工建设，大伙房输水配套工程等48个市政项目顺利实施。

——大气魄启动305国道和中华路改造提升工程，305国道两侧建成20米绿化带，中华路两侧建成30米绿化带，不仅沿路环境发生了巨大变化，而且极大地提升了城市品位和城市价值。

——全面加强生态环境建设，辽河、大辽河、大凌河60个治理项目如期竣工，造林绿化3.7万亩，节能减排完成任务，盘锦向着生态园林城市的目标加快迈进。

——以创建全国文明城市活动为抓手，全面加强交通、环境等管理，城市更加文明规范，广大市民的认同感、自豪感显著增强。

（六）民生幸福实现新提升

全市财政用于人民群众生活密切相关的民生支出123亿元，占财政总支出的66.8%。

1.就业和社会保障不断加强。新增实名制就业6.8万人，城镇登记失业率2.75%。社会保障体系进一步健全，企业退休职工养老金、居民低保补助等提标工 作全面完成，城镇基本医疗保险和大病补充保险走在全省前列。35个社区卫生服务中心和乡镇卫生院落实基本药物制度，新农合最高报销限额达到15万元，是全 省平均水平的2.5倍。

2.民生实事全面落实。农村学校全部配备安全可靠的新校车。建成农村标准化幼儿园22所。做到了不让一名学生因贫失学。建成保障性住房22361套。市中 心医院、市老干部活动中心投入使用。开工建设市社会福利中心。解决了5786户市民历史遗留的房屋产权办理问题。实施残疾人康复工程。新建城市公园9座。 维修改造街巷50条，修缮农村公路200公里。

3.社会事业全面进步。义务教育、高中阶段教育水平不断提升，学前教育、职业教育、高等教育加快发展。文化事业繁荣发展，成功举办中国盘锦国际湿地摄影展。重大疾病防控工作卓有成效，食品药品安全专项整治有力推进。全民健身运动广泛开展。

4.平安盘锦建设扎实推进。不断加强和创新社会管理，狠抓信访稳定、社会治安和安全生产，强化政府应急管理，加强人民调解和法律援助，影响社会和谐稳定的突出问题得到有效解决，信访工作目标考核继续位居全省第一，蝉联全省综治工作先进市，社会更加和谐稳定。

（七）体制机制焕发新活力

——深化行政审批制度改革，继续简政放权，市级审批项目精简到51项，我市成为辽宁权力下放最快、力度最大的城市之一。

——创新融资机制，直接融资240亿元，新增贷款113.7亿元，对上争取资金80亿元。

——创新土地开发利用管理机制，新增建设用地26平方公里，有力保障了发展用地，我市成为全国土地管理创新试点市。

——创新人才人口引进机制，加大户籍、住房、社保等政策落实力度，人才人口集聚能力大幅提升。

——创新招商体制机制，建立市直三大专业招商中心和县（区）专业招商分局，科学化、专业化的招商体系基本形成。

在推进经济社会发展的同时，着力加强民主法制建设和政府自身建设。自觉接受人大及其常委会的法律监督、工作监督和政协的民主监督，办理人大代表建议78 件、政协提案187件。大力推进依法治市、依法行政工作，加强“六五”普法。开展创先争优活动，持续推进机关效能建设，政务公开和政务服务进一步深化。加 大反腐倡廉力度，建立市公共资源交易中心，强化重点领域、关键环节的行政监察和审计监督，勤政廉政建设得到进一步加强。

国防教育、国防动员和双拥共建深入开展，人口与计划生育、民族宗教、妇女儿童、广播电视、老龄慈善、残疾人、外事侨务等事业不断发展，统计、人防、物价、质监、工商、气象、供销、住房公积金、贸促会、地方志等工作取得了新成绩。

各位代表：过去的一年，是形势复杂、困难重重的一年，也是盘锦奋发有为的一年。一年来，在市委的正确领导下，在市人大、市政协的有效监督、鼎力支持下，市 政府团结带领全市人民，满怀激情，斗志昂扬，真抓实干，用智慧、魄力和不达目的绝不罢休的精神，赢得了发展的重大胜利！

在此，我代表市人民政府，向全市各族人民，向各位人大代表、政协委员，向各民主党派、工商联、人民团体和社会各界人士，向人民解放军、武警部队官兵和辽河油田、北方华锦、辽河石化、长城钻探等驻盘单位，向所有关心支持盘锦的海内外朋友，表示诚挚的感谢！

在总结工作的同时，我们也清醒地认识到前进中存在的困难和问题：经济结构性矛盾尚未根本解决，保持经济加快增长难度加大，自主创新能力不强，资源环境和要 素约束加剧，部分群众生活困难，社会管理和公共服务水平亟待提高，政府自身建设需要不断加强。这些问题，我们将采取有效措施，切实加以解决。

二、2013年主要任务

各位代表：今年，是盘锦向更高目标迈进的关键一年。我们要以更加饱满的激情投入新的奋斗，积极应对复杂多变的经济形势，让盘锦的发展继续走在全省前列。

今年政府工作的指导思想是：全面深入学习贯彻党的十八大精神，坚持“一条主线、三大任务、四个转型”总体布局，把握扩增量、调结构、转方式、惠民生主攻方 向，以提高经济增长质量和效益为中心，以项目建设为抓手，在改革开放和创新驱动中加快辽东湾新区开发建设、加快新型产业基地建设、加快城乡一体化发展，在 转型中实现追赶，在增长中实现升级，在发展中改善民生，奋力开创向海发展全面转型以港强市新局面。

全市经济和社会发展主要预期目标：地区生产总值增长13%；固定资产投资增长25%；公共财政预算收入增长16%；社会消费品零售总额增长15%；外贸出口增长10%；利用外资达到15亿美元；城市居民人均可支配收入增长13%；农民人均纯收入增长15%。

在工作中，我们要继续实施开放战略和项目战略，狠抓招商引资，狠抓项目建设，狠抓环境建设，确保石化及精细化工、温泉旅游业和港口城市建设等实现重大突破。

（一）以更大的力度和气魄抓开放抓项目。越是形势严峻，越要加大开放力度，越要狠抓招商、狠抓项目。

1.狠抓“四个一批”。“四个一批”是项目建设的总体安排，要集中一切力量，实现项目建设大突破。

——引进一批：加大招商引资力度，引进亿元以上项目200个。

——开工一批：加大跟踪落实力度，新开工亿元以上项目200个。

——竣工一批：加大推进支持力度，竣工亿元以上项目100个。

——储备一批：高标准包装石化及精细化工、装备制造和温泉旅游、商业综合体、小商品市场等大项目200个。

2.狠抓项目服务。进一步简政放权，大力精简审批事项，建立高效快捷的办事流程，大幅提高审批效率和服务质量。各级政府都要说到做到，以信誉和服务赢得发展，建设企业家“愿意来、留得住、发展好”的最佳投资环境。

3.狠抓三大要素。

一是多渠道筹措发展资金。扩大信贷投放，新增贷款力争达到130亿元。推进企业上市、债券发行、融资租赁、融资担保等工作，鼓励民间资本进入基础设施、公用事业等领域。优化金融生态环境。

二是切实做好土地工作。抓好土地管理创新试点，实现未利用地开发新突破。加大土地增减挂钩、指标争取和存量挖潜力度，保障发展用地，同时严格规范管理，节约集约用地。

三是加大人才人口引进力度。全面落实户籍、住房、社保等各项政策，吸引更多的优秀人才，集聚更多的城市人口。加快盘锦职院新校区、华发国际学校等项目建设，以产业大发展、职业教育大发展，聚集人才，聚集人气。

（二）加快辽东湾新区建设。新区是盘锦建设世界知名港口城市的主要承载，一定要起到龙头带动作用。

1.加快建设亿吨大港。港口是盘锦崛起的关键，是“牛鼻子”工程，要举全市之力，加快港口建设。今年，港口投资要达到50亿元以上。全面推进30万吨原油 码头及25万吨航道工程。确保东港区建成10个5万吨级泊位，西港区建成4个5万吨级泊位，竣工港区铁路、油品罐区、污水处理等配套工程，吞吐能力达到 4000万吨。全面提升港口通关效率和服务功能，晋升为国家一类口岸。

2.加快做大产业规模。产业是新区发展的核心支撑，要以第一位的精力抓招商、抓项目。开工建设总投资845亿元的兵器集团精细化工及原料工程，推进长春石 化、联成化学、忠旺铝材、合力叉车等项目取得重大进展，引进10亿元以上项目22个。石化及精细化工、临港先进装备制造两大主导产业要尽快上规模、上水 平。

3.加快水城建设。确保大连理工大学盘锦校区交付使用，竣工行政文化中心、体育中心、盛京医院辽东湾分院等重大工程。加快建设路网、绿网、水网等公共设施，推进新城全面开发，集聚人气商气。

今年，第十二届全运会青年女子足球赛和青年男子排球赛将在新区举行，我们一定全力办好两项赛事，让全国人民看到盘锦的风采！

（三）推进工业大发展、快发展。发展石化及精细化工、先进装备制造业是盘锦工业的两大主题，关系着盘锦的未来，必须抓实抓好。

1.加快建设世界级石化及精细化工产业基地。全面落实《盘锦市建设世界级石化及精细化工产业基地规划纲要》，打造中国的“裕廊”。

——园区建设要有大提升。抓好辽东湾和兴隆台、双台子、盘山4个重点化工园区，全面提升园区承载和服务能力，发展基本有机化工、精细化工、化工新材料等产业，延长产业链，提高产业丰厚度。

——招商引资要有大突破。全力引进一批具有世界级技术水平的大企业，重点抓好台湾地区招商，让盘锦成为台湾精细化工产业转移的主要承接地。引进2亿元以上项目45个，其中10亿元以上项目15个。

——大企业大项目要有大进展。全力支持辽河油田和北方华锦，推进辽河石化汽柴油加氢、北沥润滑油基础油、宝来石化芳烃合成、和运异戊橡胶、新和成蛋氨酸、 胜友顺丁橡胶等30个重点项目加快建设。力争5年，形成主营业务收入10亿元以上企业30家，其中100亿元以上企业8家，1000亿元以上企业2家。

2.加快建设国内领先的先进装备制造业基地。编制实施装备制造业发展规划，发展石油天然气装备、海洋工程装备、重型装备等先进装备制造业。大力建设辽东 湾、大洼临港、兴隆台3个装备制造业基地，确保航天晨光化工装备、奥德燃气装备等项目开工建设，推进辽河重工海工基地、长城钻探工业基地等项目加快建设， 引进2亿元以上项目25个。

3.全面提升工业整体竞争力。狠抓工业五项工程，推进企业改造提升和并购，抓好50个重点项目，狠抓节能降耗和落后产能淘汰，工业产业集群主营业务收入达 到2000亿元。以科技创新引领产业发展，加快建设和运橡胶、辽宁陆海、辽河石化等企业研发机构，推进重大创新工程，高新技术产品增加值增长20%以上。 加快形成公共研发、检测等服务平台。抓好标准化厂房建设。大力支持民营经济，培育创新型小微企业。

（四）建设具有吸引力的国际旅游目的地。红海滩、芦苇荡是世界最大的滨海湿地，是独一无二的生态旅游资源。以旅游业为龙头，拉动服务业大发展、快发展。

1.搭建服务业发展平台。

——旗舰型服务业集聚区：以305国道、中华路、向海大道为支撑，集聚服务业大项目，建设盘锦的“金廊银带”。

——三大旅游载体：全力建设红海滩、辽河口旅游度假区和大洼温泉新城，以此为龙头，做大温泉旅游产业。

——城市CBD：重点发展精品商业、高端商业，建设10平方公里兴隆台中央商务区。

——县区服务业集聚区：每个县区和经济区规划建设1个5-10平方公里的现代服务业集聚区。

2.发展壮大特色服务业。

一是大力发展温泉旅游业。推进天沐温泉城、海泉湾温泉度假中心，万豪、喜来登、铂尔曼五星级酒店等项目加快建设，确保富盈温泉城、中惠源温泉等项目开工建设，建设具有吸引力的国际旅游目的地。

二是大力发展文化产业。推进文化与旅游融合发展，加快甜水湖东方文化旅游城、志高动漫科技产业园、北方欢乐世界主题公园、锦联不夜城等项目建设，打造一批文化旅游精品，建设辽宁重要的文化产业基地。

三是大力发展高端商业。推进东方银座、华联、佳兆业、兴隆大家庭等项目加快建设，确保华氏、泛华城市综合体等项目开工建设，以兴隆台中央商务区为重点，建设继沈阳、大连之后的辽宁第三大购物中心。

四是大力推进商品交易市场建设。推进红星国际广场、蓝碧源五金机电汽配城、天龙汽配城和中国鲜活水产城加快建设，确保新湖中宝国际商贸城、翔球商贸城等项目开工建设，建设东北重要的商品交易集散地。

五是大力发展会展业。推进丰亿、瑞唐、泛华和富盈会展中心加快建设，办好湿地旅游周等展会活动，发展会议经济、会展经济，建设辽宁有影响力的会展城市。

六是大力发展现代物流业。以港口为龙头，推进益海嘉里物流、传化双益公路港等项目加快建设，做大化工物流、粮食物流、装备物流、邮政物流等规模，建设东北重要的物流枢纽城市。

3.稳步推进房地产业发展。全面落实国家调控政策，大力推进房地产结构调整，重点发展生态高端地产，发展温泉地产、旅游地产、商业地产、教育地产、养生地产等可持续地产项目，力争房地产投资增长25%、商品房销售额增长15%，建设区域性的生态居住中心。

（五）大力发展现代农业。农业是安天下、稳民心的战略性基础产业，要进一步加大投入，促进农业增效、农民增收。

1.大力推进农业对外开放。多渠道吸引国内外资本，引进10亿元以上项目3个。推进农业产业化、规模化、市场化，突出抓好田园现代农业产业园、德胜丰润农产品加工、宝迪食品加工、正邦生猪养殖加工等重点项目。加快建设高升国家级现代农业综合示范区。

2.大力发展生态高效农业。加大资源整合和精品培育力度，开工建设香港宏安河蟹市场，办好北京盘锦大米、河蟹展销等活动，发展壮大盘锦大米、河蟹两大龙头 产业。继续推进“一县一业”，加大土地流转力度，抓好设施农业、苇田生态养殖、畜禽养殖等项目建设，力争大洼成为辽宁“一县一业”示范县。

3.大力加强农业基础建设。严格耕地保护，大兴农田水利，提高农业抗灾减灾能力，稳定粮食生产。加强动植物疫病防治、农产品质量检测、农技推广体系建设，实施万亩林地养护工程，发展社会化农技与服务组织，进一步扩大农业保险覆盖面，提高农业机械化水平。

（六）建设城乡一体化发展示范市。以“5城7镇”为重点，加快城镇化步伐，推进城乡一体化发展。

1.加大城镇建设力度。兴隆台要在中央商务区和东部新城建设上实现重大突破，双台子要继续加大辽河新城开发和老城改造力度，盘山新县城建设要上新台阶，大 洼要确保温泉新城建设取得重大进展，同时推进7个中心镇加快建设。妥善解决进城农民户籍、就业、社保等问题，有序推进农业转移人口市民化。今年，城镇化要 有新进展、新亮点！

2.加快基础设施建设。实施10项重点交通工程，确保疏港铁路正式运营，辽东湾互通立交、营盘客专、阜盘高速、兴辽路、林丰路建成通车，抓好沈盘铁路、滨 海公路、疏港高速，改造维修农村公路，推进城市道路工程。确保华润城市热电投入使用，竣工大伙房输水配套工程，推进农村居民安全饮水、城市基础设施配套等 工程建设。

3.切实加强城市管理。进一步加大全国文明城市创建力度，大力推进道路交通秩序、市容环境、市场秩序等综合整治。加强城市精细化管理，推进数字城市建设，让城市管理再上一个新台阶。

4.着力提升生态建设水平。全面推进生态县、生态镇和生态园区创建工作。实施“蓝天工程”，抓好绿色交通、大气监控等措施，推进民用燃气网络向乡镇延伸。 巩固辽河流域阶段性治理成果，加强污水处理设施建设，强化沿河村屯环境整治。加强湿地和海洋保护，重点抓好湿地水利设施建设。造林绿化700万平方米以 上，建成华发、辽河生态等8座公园，推进生态园林城市建设。

各位代表：生态环境事关子孙后代，事关人民福祉。我们不仅要加快发展，更要加强生态建设，让天更蓝、水更清、地更绿，建设美丽盘锦！

（七）加强以保障和改善民生为重点的社会建设。

1.大力提升就业和社会保障能力。完善就业培训等服务体系，新增就业7万人，城镇登记失业率控制在3%以下。做好城乡居民养老保险金补助、居民低保、新农 合补助等提标工作，抓实基本医疗保险和大病保险。加快提高低收入群众收入水平。继续推进棚户区、城中村、城边村、老旧弃管小区等改造工程，加快保障性安居 工程建设，着力解决城区和县城冷楼供暖问题。

2.推进社会事业全面进步。优先发展教育，优化资源布局，协调发展各级各类教育，建设基础教育强市。提高基层卫生机构服务水平，抓好公立医院改革试点，启 动建设精神疾病专科医院。积极开展文艺精品创作等活动，丰富群众文化生活。推进国防和民兵预备役建设，加强双拥共建，全面做好人口与计划生育、民族宗教、 防震减灾、妇女儿童、老龄慈善、残疾人等工作。

3.加强和创新社会管理。建立街道和乡镇社会管理服务中心，健全基层组织服务功能。积极构建和谐劳动关系。完善“大调解”体系，依法处理信访事项，切实维 护群众合法权益。落实和完善安全生产制度，杜绝重特大事故发生。严格食品药品安全监管。提高突发事件应急处置能力。扎实做好 “六五”普法工作。强化社会管理综合治理，确保社会大局和谐稳定。

民生连着发展，连着民心。我们要始终把人民放在心中最高位置，为群众办好事、办实事、办真事，让全市人民享受到实实在在的发展成果。

各位代表，按照全省统一部署，今年是本届市政府任期的最后一年。几年来，我们始终恪守“为民、务实、高效、清廉”的执政理念，尽心竭力，不敢懈怠，在市委 的领导下，办成了一些事关长远的大事，办好了一些关乎民生的实事，破解了一些影响和谐的难事。面对新的形势、新的任务，我们要进一步加强政府自身建设。

我们要做到依法行政：自觉接受人大及其常委会的法律监督、工作监督和政协民主监督，虚心听取各方面意见，不断改进政府工作。坚持科学民主决策，推进政务公开，保证各项决策符合人民意愿，经得起实践检验。

我们要做到真抓实干：始终把任务和目标抓在手上，困难面前不退缩，矛盾面前不推诿，敢于担当，敢抓敢管，以解决问题为核心确保工作落实，以业绩论英雄。

我们要做到高效服务：把提高工作效率作为机关建设的第一要务，转变作风，少开会、开短会，讲短话、讲实话，以等不起的紧迫感、慢不得的责任感，为民造福。

我们要做到锐意创新：把改革创新贯穿政府工作的始终，坚持解放思想，勇于探索，大胆实践，用创新的思维、改革的办法解决新矛盾、新问题。

我们要做到廉洁从政：牢固树立艰苦奋斗、勤俭节约的思想，严格执行党风廉政建设规定，规范行政行为，力戒奢靡之风，反对铺张浪费，努力建设人民满意的政府。

各位代表，新的一年已经开始，我们踏上了充满希望的新征程。让我们在市委的领导下，万众一心，开拓奋进，真抓实干，共同创造盘锦更加美好的明天！
